# Association between hematocrit and cranial MRI abnormalities in neonatal hyperbilirubinemia

**DOI:** 10.3389/fped.2026.1785949

**Published:** 2026-05-28

**Authors:** Ting Liu, Liang Wang, Rufeng Ji, Hongjuan Wei

**Affiliations:** 1 Department of Pediatric, Nanjing Lishui People's Hospital, Zhongda Hospital Lishui Branch, Southeast University, Nanjing, China; 2 Department of Medical Imaging, Nanjing Lishui People's Hospital, Zhongda Hospital Lishui Branch, Southeast University, Nanjing, China

**Keywords:** biomarker, brain disease, hematocrit, magnetic resonance imaging, neonatal hyperbilirubinemia

## Abstract

**Background:**

In the context of neonates, the association between hematocrit (HCT) and cranial MRI abnormalities continues to be a topic of controversy. At present, the available evidence regarding the relationship between HCT and cranial MRI abnormalities is inadequate.

**Objective:**

This study aims to elucidate the relationship between HCT and cranial MRI abnormalities in neonatal hyperbilirubinemia (NHB).

**Methods:**

We conducted a retrospective cross-sectional study of 410 neonates with hyperbilirubinemia. Neonatal blood parameters, maternal prenatal data, and cranial MRI findings were extracted from the electronic medical record system. Logistic regression and smooth curve fitting were used to analyze the associations.

**Results:**

After adjusting for confounding factors, multivariate logistic regression analysis showed that each 1% increase in HCT is associated with a 6% increase in the risk of cranial MRI abnormalities. Further exploratory subgroup analyses based on sex, mode of delivery, and GDM revealed no significant interactions between these subgroups (all *P* for interaction > 0.05).

**Conclusions:**

Among NHB, higher HCT was significantly associated with higher risk of incident cranial MRI abnormalities. These findings suggest that HCT may serve as a potential risk factor for cranial MRI abnormalities and could be a relevant biomarker.

## Introduction

Neonatal hyperbilirubinemia (NHB) affects 60%–80% of term newborns and is a leading cause of hospitalization in the first week of life (1). Although most cases resolve without sequelae, severe NHB can cause bilirubin-induced neurological dysfunction, leading to long-term motor, cognitive, and auditory impairments (2). Cranial magnetic resonance imaging (MRI) is a sensitive tool for detecting brain injury in neonates; abnormalities include hemorrhagic, ischemic, and structural lesions (3). In our cohort, 26.3% of neonates with severe hyperbilirubinemia exhibited such MRI abnormalities, underscoring the need for early risk stratification. Hematocrit (HCT) is a routine laboratory parameter. In term neonates, normal HCT at birth ranges from approximately 45%–65% (4). HCT directly determines blood viscosity and oxygen-carrying capacity. Elevated HCT increases viscosity, impairs cerebral microcirculation, and may reduce oxygen delivery (5), while low HCT compromises oxygen transport (6). HCT reflects the concentration of red blood cells, which is relatively stable over short intervals as the mean neonatal RBC lifespan is 54.2 ± 11.3 days (7). However, rapid and significant hematocrit shifts can occur following hemolysis or transfusion (8). Although cranial ultrasound is often used as a first-line screen in NHB, it has limited sensitivity for subtle parenchymal lesions. MRI offers superior diagnostic accuracy (9). A simple biomarker such as HCT could help refine candidate selection for neuroimaging, yet direct evidence linking HCT to cranial MRI abnormalities in NHB is lacking. Therefore, this study aimed to investigate the relationship between admission HCT and cranial MRI abnormalities in a retrospective cohort of neonates with hyperbilirubinemia, to improve risk stratification and guide targeted neuroimaging.

## Materials and methods

### Study population

**Figure 1:**
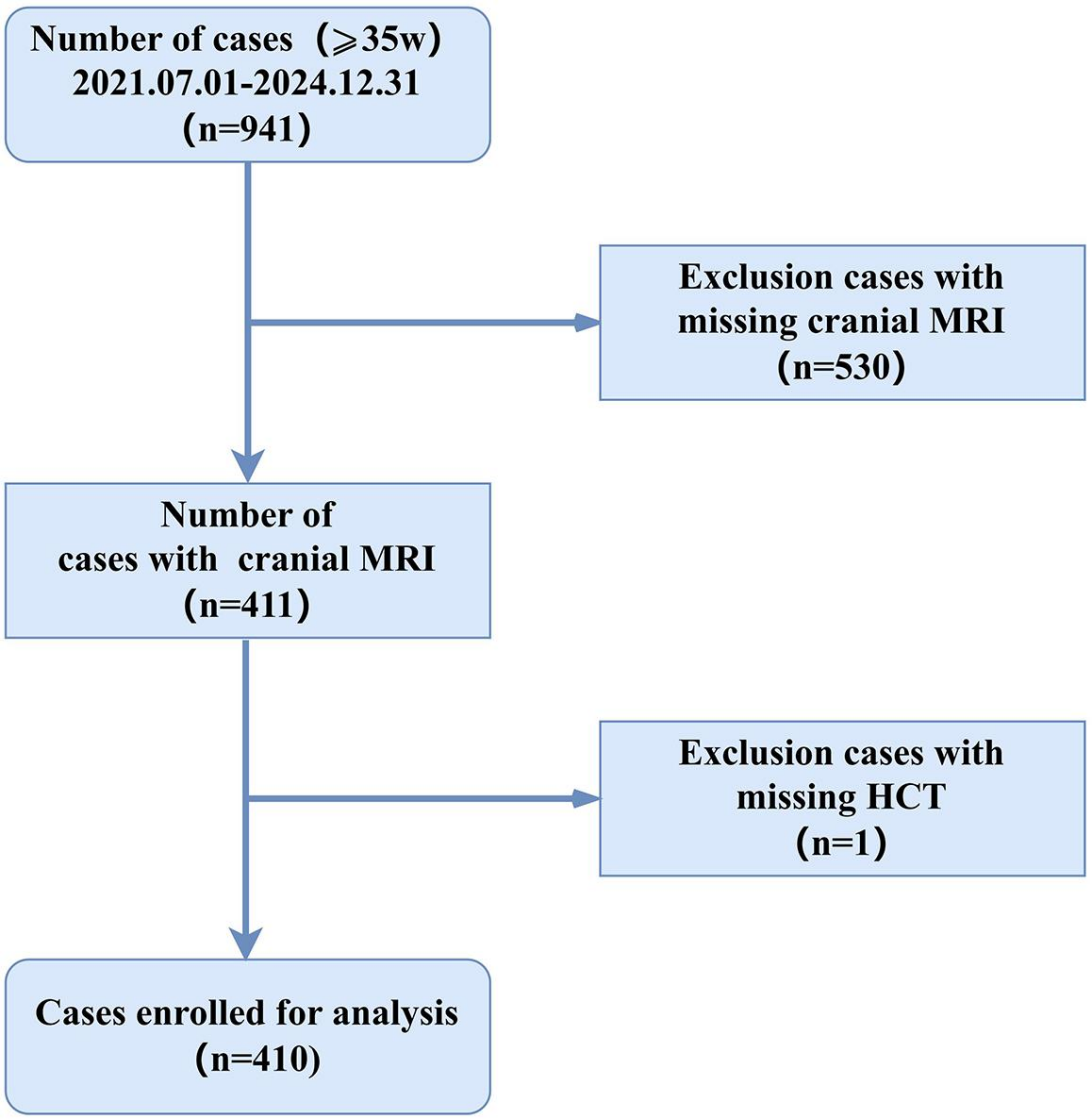
Flowchart for the study population.

This study adhered to the STROBE guidelines for observational research. We conducted a retrospective cross-sectional analysis of 941 late-preterm and term neonates (≥35 weeks’ gestation) with hyperbilirubinemia requiring NICU admission at Nanjing Lishui People’s Hospital (NJLSPH) from July 1, 2021 to December 31, 2024. Clinical data were extracted from the institutional electronic medical records. Neonates with missing HCT or cranial MRI results were excluded. Ultimately, 410 patients were included in the final analysis (Figure 1). The study protocol received ethical approval from the NJLSPH Research Ethics Committee (Approval No. 2025KY0424-03). Due to the retrospective nature of the study and the use of fully anonymized data, the ethics committee waived the requirement for written informed consent. This study was registered at the Chinese Clinical Trial Registry Center (Registration Number: ChiCTR2500101885).

### Laboratory, MRI data collection and measures

Data were retrospectively collected from neonates with NHB admitted to NJLSPH between July 1, 2021 and December 31, 2024. The database comprised demographic characteristics, laboratory parameters, and neuroimaging findings. Neonatal demographic characteristics included sex, birth weight, admission weight, age, blood type, and other relevant factors. Maternal demographic variables included gestational week, age, blood type, gravidity, parity, and mode of delivery. Laboratory parameters consisted of hematologic indices (WBC, NEUT%, LYMPH%, RBC, HGB, HCT, MCV, MCH, RDW-CV, PLT), inflammatory markers (hsCRP, PCT), and hepatic function (TBIL, ALT, AST, GGT, ALP, ALB, GLO). Clinical biochemical parameters (including TBIL, ALT, GGT, ALP, ALB, GLO) were quantified utilizing an automated clinical chemistry analyzer (AU5800; Beckman Coulter Trading Co. Ltd., China). Hematologic indices (hsCRP, WBC, RBC, HGB, HCT, MCV, MCH, RDW-CV) were measured using a hematology analyzer (BC-7500; Mindray Corporation, Shenzhen, China). All laboratory variables, including HCT, were measured within 2 h of admission and before phototherapy. Thus, HCT measurement preceded or coincided with cranial MRI in all cases. Cranial magnetic resonance imaging (MRI) was performed on a 3.0 T scanner, acquiring the following sequences: T1-weighted imaging (T1WI), T2-weighted imaging (T2WI), T2-fluid-attenuated inversion recovery (T2-FLAIR), T2-propeller (T2-Prop), diffusion-weighted imaging (DWI), apparent diffusion coefficient mapping (ADC), exponential apparent diffusion coefficient mapping (eADC), and susceptibility-weighted angiography (SWAN). During the neonatal hospitalization period, cranial MRI was performed in cases with serum bilirubin levels significantly elevated to or above 342 µmol/L (10), abnormal cranial ultrasound screening results, or in the presence of high-risk factors such as *in utero* cerebral developmental anomalies. MRI findings were categorized into two groups: normal and abnormal. MRI abnormalities included hemorrhagic lesions, ischemic lesions, choroid plexus cysts, venous malformations, neuroepithelial cysts, gray-white matter heterotopia, ventricular enlargement, hydrocephalus, etc. Abnormalities on cranial MRI do not include cephalohematomas that are detectable by visual inspection or palpation. (Table 1, Supplementary Figures S1, 2).

**Table 1:** Baseline characteristics of the study population.

Variables	Total (*n* = 410)	Non-Cranial MRI abnormality (*n* = 302)	Cranial MRI abnormality (*n* = 108)	*P*	Statistic
Diagnosis, *n* (%)				0.512	0.429
Hyperbilirubinemia	357 (87.1)	261 (86.4)	96 (88.9)		
ABO hemolytic jaundice	53 (12.9)	41 (13.6)	12 (11.1)		
Sex, *n* (%)				0.619	0.247
Male	227 (55.4)	165 (54.6)	62 (57.4)		
Female	183 (44.6)	137 (45.4)	46 (42.6)		
Birth weight, g	3366.4 ± 445.8	3369.2 ± 442.6	3358.4 ± 456.3	0.829	0.047
Admission weight, g	3187.6 ± 447.4	3183.6 ± 443.4	3198.5 ± 460.2	0.767	0.088
Neonatal blood type, *n* (%)				0.611	Fisher
O+	128 (31.4)	95 (31.7)	33 (30.6)		
A+	137 (33.6)	103 (34.3)	34 (31.5)		
B+	116 (28.4)	84 (28.0)	32 (29.6)		
AB+	25 (6.1)	17 (5.7)	8 (7.4)		
O-	1 (0.2)	1 (0.3)	0 (0)		
AB-	1 (0.2)	0 (0)	1 (0.9)		
Urine PH, *n* (%)				0.486	Fisher
5	49 (12.7)	39 (13.5)	10 (10.3)		
5.5	78 (20.2)	59 (20.4)	19 (19.6)		
6	120 (31.1)	83 (28.7)	37 (38.1)		
6.5	105 (27.2)	79 (27.3)	26 (26.8)		
7	32 (8.3)	27 (9.3)	5 (5.2)		
7.5	2 (0.5)	2 (0.7)	0 (0)		
Cranial MRI, *n* (%)				<0.001	Fisher
No abnormality	302 (73.7)	302 (100)	0 (0)		
Hemorrhagic lesion	76 (18.5)	0 (0)	76 (70.4)		
Ischemic lesion	15 (3.7)	0 (0)	15 (13.9)		
Hemorrhagic and ischemic lesion	4 (1.0)	0 (0)	4 (3.7)		
Other abnormality	13 (3.2)	0 (0)	13 (12.0)		
Cranial ultrasonography, *n* (%)				<0.001	Fisher
No abnormality	241 (70.5)	199 (76.5)	42 (51.2)		
Subependymal cyst	57 (16.7)	42 (16.2)	15 (18.3)		
Choroidal cyst	14 (4.1)	8 (3.1)	6 (7.3)		
Ventricular enlargement	5 (1.5)	3 (1.2)	2 (2.4)		
Cephalohematoma	9 (2.6)	4 (1.5)	5 (6.1)		
Intracranial hemorrhage	10 (2.9)	2 (0.8)	8 (9.8)		
Subependymal cyst and ventricular enlargement	3 (0.9)	1 (0.4)	2 (2.4)		
Subependymal cyst and intracranial hemorrhage	1 (0.3)	0 (0)	1 (1.2)		
Subependymal cyst and cephalohematoma	1 (0.3)	1 (0.4)	0 (0)		
Choroidal cyst and ventricular enlargement	1 (0.3)	0 (0)	1 (1.2)		
hsCRP, mg/L	1.2 (0.8, 3.5)	1.1 (0.8, 3.3)	1.6 (0.8, 4.7)	0.155	2.020
RBC, 10^12^/L	5.0 ± 0.6	4.9 ± 0.6	5.1 ± 0.6	0.064	3.462
HGB, g/L	174.3 ± 19.9	173.2 ± 20.2	177.2 ± 18.8	0.072	3.263
HCT, %	49.7 ± 6.0	49.3 ± 6.0	50.9 ± 5.6	0.019	5.544
MCV, fL	100.1 ± 4.8	100.0 ± 4.7	100.6 ± 5.0	0.271	1.215
MCH, pg	35.1 ± 1.8	35.1 ± 1.8	35.0 ± 1.7	0.602	0.273
MCHC, g/L	350.9 ± 13.2	351.6 ± 13.3	348.7 ± 12.6	0.045	4.028
RDW-CV, %	15.3 ± 1.1	15.3 ± 1.1	15.2 ± 1.1	0.314	1.018
RDW-SD, fL	55.6 ± 4.0	55.6 ± 4.2	55.6 ± 3.5	0.880	0.023
WBC, 10^9^/L	10.8 ± 3.3	10.9 ± 3.4	10.6 ± 2.8	0.366	0.819
NEUT, 10^9^/L	4.9 ± 2.5	4.9 ± 2.6	5.0 ± 2.4	0.645	0.212
LYMPH, 10^9^/L	4.2 ± 1.3	4.3 ± 1.4	3.9 ± 1.3	0.008	7.072
MONO, 10^9^/L	1.1 ± 0.4	1.1 ± 0.4	1.1 ± 0.4	0.826	0.048
EO, 10^9^/L	0.4 (0.3, 0.5)	0.4 (0.3, 0.5)	0.4 (0.3, 0.5)	0.533	0.388
PLT, 10^9^/L	279.7 ± 80.5	283.1 ± 83.9	270.3 ± 69.6	0.158	2.002
PCT, %	0.3 ± 0.1	0.3 ± 0.1	0.3 ± 0.1	0.055	3.704
MPV, fL	10.1 ± 1.0	10.1 ± 1.0	9.9 ± 0.9	0.049	3.913
PDW, fL	14.9 ± 2.3	14.8 ± 2.4	15.2 ± 2.2	0.092	2.848
*P*-LCR, %	26.6 ± 6.4	26.8 ± 6.5	25.8 ± 6.1	0.173	1.864
First TBIL, μmol/L	328.3 ± 53.2	325.9 ± 53.4	334.9 ± 52.2	0.132	2.277
ALT, U/L	16.8 ± 6.4	16.7 ± 6.5	16.8 ± 6.2	0.968	0.002
AST, U/L	48.0 ± 29.7	47.2 ± 32.1	50.1 ± 21.3	0.382	0.767
TP, g/L	60.0 ± 5.6	59.8 ± 5.5	60.5 ± 6.1	0.292	1.114
ALB, g/L	36.7 ± 3.2	36.6 ± 3.3	37.0 ± 3.2	0.362	0.831
GLO, g/L	23.3 ± 4.0	23.2 ± 3.9	23.5 ± 4.3	0.458	0.553
A/G	1.6 ± 0.4	1.6 ± 0.3	1.6 ± 0.4	0.835	0.043
GGT, U/L	156.1 ± 67.2	153.8 ± 64.7	162.7 ± 73.8	0.235	1.412
ALP, U/L	162.5 ± 58.0	162.4 ± 56.3	162.9 ± 62.6	0.937	0.006
GLU, mmol/L	4.1 (3.6, 4.8)	4.2 (3.6, 4.9)	4.0 (3.6, 4.6)	0.162	1.952
CK-MB, U/L	41.5 ± 20.7	40.7 ± 21.7	43.8 ± 17.4	0.186	1.759
K^+^, mmol/L	4.9 ± 0.5	4.9 ± 0.5	4.9 ± 0.5	0.509	0.436
Na^+^, mmol/L	139.8 ± 3.3	139.6 ± 3.2	140.3 ± 3.5	0.089	2.912
Cl^−^, mmol/L	106.7 ± 3.4	106.6 ± 3.4	107.1 ± 3.5	0.184	1.770
Maternal age, year	29.7 ± 4.4	30.0 ± 4.5	28.9 ± 4.1	0.042	4.175
Education, (%)				0.678	Fisher
Elementary school	5 (1.3)	4 (1.4)	1 (1.0)		
Junior high school	47 (12.6)	37 (13.4)	10 (10.3)		
High school	26 (7.0)	21 (7.6)	5 (5.2)		
Junior college	52 (13.9)	40 (14.4)	12 (12.4)		
College	116 (31.0)	82 (29.6)	34 (35.1)		
Bachelor	122 (32.6)	87 (31.4)	35 (36.1)		
Master + ^a^	6 (1.6)	6 (2.2)	0 (0)		
Ethnicity, *n* (%)				1	Fisher
Han chinese	372 (98.9)	274 (98.6)	98 (100)		
Other	4 (1.1)	4 (1.4)	0 (0)		
Week of gestation, week	38.9 ± 1.3	39.0 ± 1.3	38.8 ± 1.3	0.201	1.640
Gravidity, *n* (%)				0.091	Fisher
1	196 (47.8)	131 (43.4)	65 (60.2)		
2	102 (24.9)	80 (26.5)	22 (20.4)		
3	53 (12.9)	41 (13.6)	12 (11.1)		
4	36 (8.8)	30 (9.9)	6 (5.6)		
5	13 (3.2)	11 (3.6)	2 (1.9)		
6 + ^b^	10 (2.4)	9 (3.0)	1 (0.9)		
Parity, *n* (%)				0.013	Fisher
1	242 (59.0)	165 (54.6)	77 (71.3)		
2	147 (35.9)	118 (39.1)	29 (26.9)		
3	19 (4.6)	17 (5.6)	2 (1.9)		
4	2 (0.5)	2 (0.7)	0 (0)		
Delivery mode, *n* (%)				< 0.001	Fisher
Vaginal delivery	279 (68.0)	183 (60.6)	96 (88.9)		
Cesarean section	125 (30.5)	113 (37.4)	12 (11.1)		
Vaginal delivery to cesarean section	6 (1.5)	6 (2.0)	0 (0)		
Mother blood type, *n* (%)				0.632	Fisher
O+	165 (41.2)	126 (42.7)	39 (37.1)		
A+	95 (23.8)	68 (23.1)	27 (25.7)		
B+	109 (27.3)	76 (25.8)	33 (31.4)		
AB+	29 (7.2)	23 (7.8)	6 (5.7)		
O-	2 (0.5)	2 (0.7)	0 (0)		
Pre-pregnancy BMI, kg/m^2^	22.7 ± 3.7	22.8 ± 3.8	22.2 ± 3.4	0.154	2.045
Mother present weight, kg	73.4 ± 10.9	73.6 ± 11.1	73.1 ± 10.2	0.679	0.171
GDM, *n* (%)				0.007	7.298
No	297 (72.4)	208 (68.9)	89 (82.4)		
Yes	113 (27.6)	94 (31.1)	19 (17.6)		
HDP, *n* (%)				0.102	2.667
No	371 (90.5)	269 (89.1)	102 (94.4)		
Yes	39 (9.5)	33 (10.9)	6 (5.6)		
Hypothyroidism, *n* (%)				0.565	0.332
No	366 (89.3)	268 (88.7)	98 (90.7)		
Yes	44 (10.7)	34 (11.3)	10 (9.3)		
ICP, *n* (%)				0.490	Fisher
No	399 (97.3)	295 (97.7)	104 (96.3)		
Yes	11 (2.7)	7 (2.3)	4 (3.7)		
Anemia, *n* (%)				0.698	0.151
No	380 (92.7)	279 (92.4)	101 (93.5)		
Yes	30 (7.3)	23 (7.6)	7 (6.5)		
IVF, *n* (%)				0.681	Fisher
No	403 (98.3)	296 (98.0)	107 (99.1)		
Yes	7 (1.7)	6 (2.0)	1 (0.9)		
Twin, *n* (%)				1	Fisher
No	408 (99.5)	300 (99.3)	108 (100)		
Yes	2 (0.5)	2 (0.7)	0 (0)		
1-min Apgar, *n* (%)				0.411	Fisher
7	2 (0.5)	2 (0.7)	0 (0)		
8	7 (1.9)	7 (2.5)	0 (0)		
9	9 (2.4)	7 (2.5)	2 (2.0)		
10	359 (95.2)	263 (94.3)	96 (98.0)		
5-min Apgar, *n* (%)				1	Fisher
9	1 (0.3)	1 (0.4)	0 (0)		
10	376 (99.7)	278 (99.6)	98 (100)		
Neonatal age, hour	125.7 ± 74.0	133.4 ± 78.0	104.1 ± 56.5	<0.001	12.814
Hospitalization duration, hour	103.0 ± 22.1	103.2 ± 20.0	102.5 ± 27.3	0.790	0.071

Data presented are mean ± standard deviation description, quartile description, or *n* (%).

a Master+: Including master's degree and higher.

b 6+: Including pregnancy 6 times and above.

### Statistical analysis

All statistical analyses were conducted using R Statistical Software (V4.2.2; R Foundation) and the Free Statistics analysis platform (Version 2.1, Beijing, China, http://www.clinicalscientists.cn/freestatistics). Statistical significance was defined as a two-sided *P*-value < 0.05. Histogram distributions, Q-Q plots, and the Kolmogorov–Smirnov test were employed to assess the normality of variable distributions. Normally distributed continuous variables were expressed as mean ± standard deviation (SD), while skewed continuous variables were represented as median with interquartile range (IQR). Categorical variables were presented as frequencies and percentages (%). Comparisons of continuous variables among groups were conducted using either the independent samples Student’s t-test or the Mann–Whitney U-test, depending on the distribution’s normality. Categorical data were compared using the chi-square test or Fisher’s exact test as appropriate. We used logistic regression to investigate the associations between HCT and cranial MRI abnormalities in NHB. HCT was entered as a continuous variable (per 0.01 unit) and as a categorical variable (quartiles). We selected these confounders on the basis of clinical interest, previous scientific literature, significant covariates in the univariate analysis, or a ≥ 10% change in the effect estimate upon their inclusion in the model. We constructed three hierarchical regression models to sequentially account for potential confounding factors: Model I adjusted for basic neonatal characteristics (age, sex, birth weight, admission weight, and gestational week); Model II additionally incorporated maternal characteristics (maternal age, pre-pregnancy BMI, and delivery mode); Model III further included neonatal hematological parameters (WBC and PLT) to address residual confounding. Tests for trend were conducted with multivariate regression models by entering the median value of each quartile as a continuous variable in the models. We employed restricted cubic splines to model potential nonlinear associations between HCT and cranial MRI abnormalities in neonatal hyperbilirubinemia. HCT was parameterized as a continuous predictor with four prespecified knots at the 5th, 35th, 65th, and 95th percentiles according to standard recommendations (11). Non-linearity was tested by including a quadratic term in the regression models. Subgroup analyses stratified by sex, delivery mode, and GDM evaluated the association between HCT and cranial MRI abnormalities in NHB infants.

## Results

### Population characteristics

A total of 410 neonates with NHB who underwent cranial MRI were included in the final analysis. Among these, 108 (26.3%) were diagnosed with cranial MRI abnormalities. As shown in Table 1, neonates with MRI abnormalities exhibited significantly higher HCT (50.9 ± 5.6% vs. 49.3 ± 6.0%, *P* = 0.019), and higher rates of vaginal delivery (88.9% vs. 60.6%, *P* < 0.001) compared to those without abnormalities. However, no statistically significant intergroup differences were observed in sex distribution, birth weight, gestational age, or most maternal characteristics (all *P* > 0.05).

### Association between HCT and cranial MRI abnormalities

**Figure 2:**
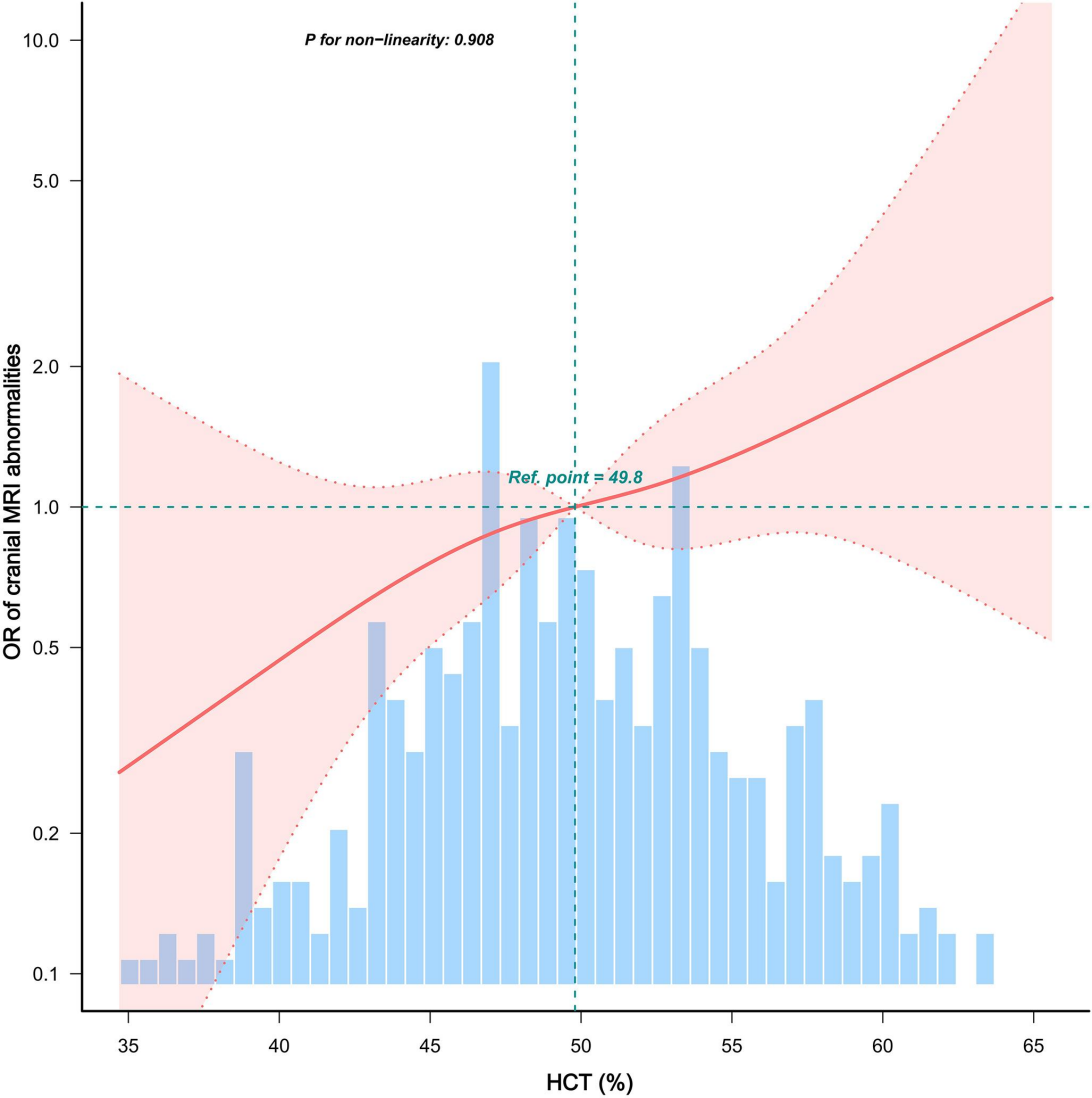
Linear dose-response relationship between HCT and *OR* of cranial MRI abnormalities. The pink solid line indicates multivariate adjusted OR and the pink dashed lines indicate the 95% CI derived from restricted cubic spline regression. The blue histogram represents the frequency distribution of HCT values across the study population. Knots are located at the 5th, 35th, 65th, and 95th percentiles for HCT. The horizontal dotted lines represent the OR of 1.0 (reference point). The reference point was set at the median level of HCT (49.8%). The logistic regression was adjusted for age, sex, birth weight, admission weight, week of gestation, maternal age, pre-pregnancy BMI, delivery mode, WBC, and PLT.

**Figure 3:**
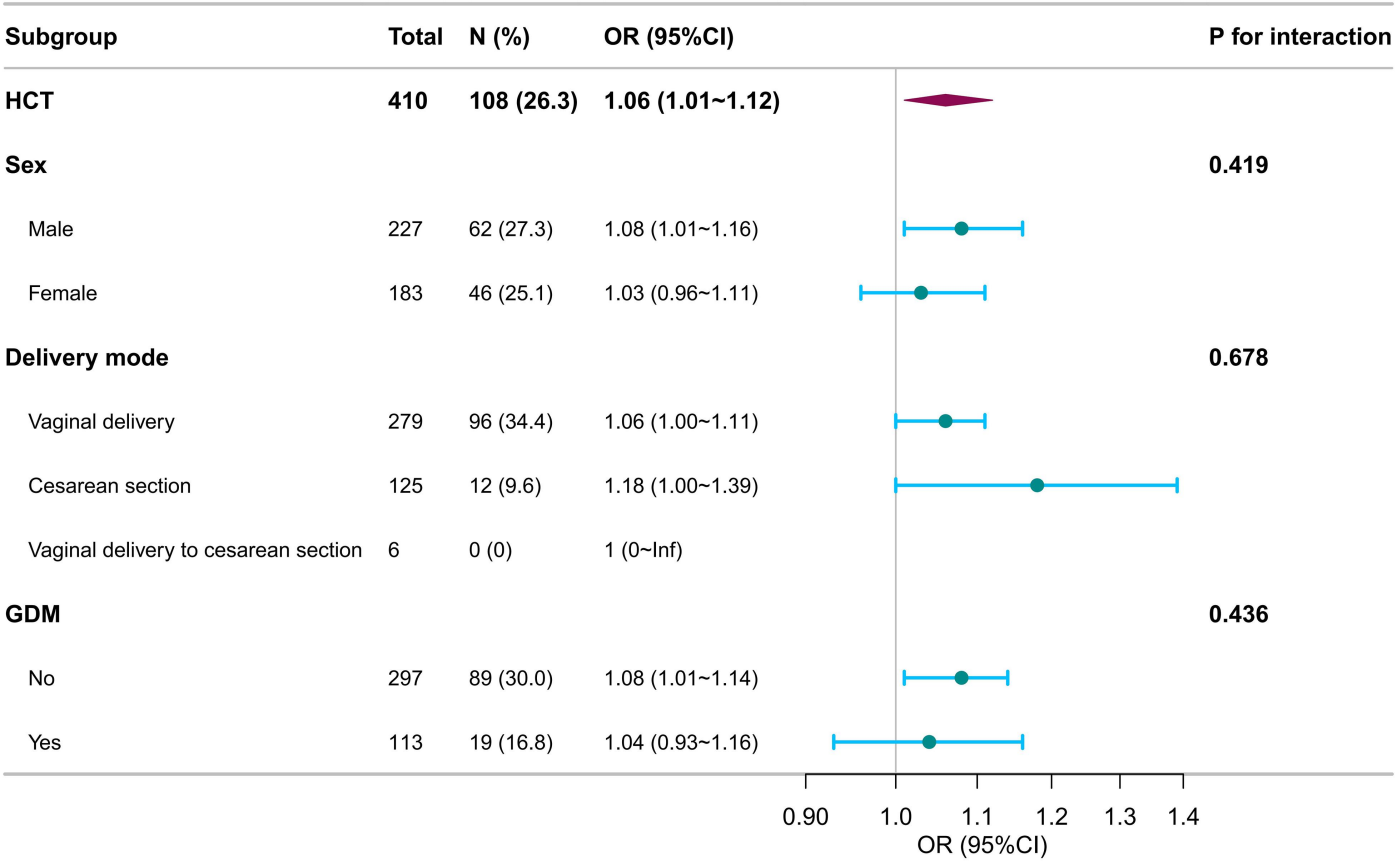
Forest plot of subgroup analyses for the association between HCT and cranial MRI abnormalities in NHB. The purple diamond represents the OR and 95% CI for each 1% increase in HCT (continuous variable) on the risk of cranial MRI abnormalities.The green dots with blue lines display the stratified OR and 95% CI for each subgroup (sex, delivery mode, and GDM). Subgroup stratification includes sex (male vs. female), delivery mode (vaginal delivery vs. cesarean section), and GDM (no vs. yes). Results are derived from Model III, adjusted for age, sex, birth weight, admission weight, week of gestation, maternal age, pre-pregnancy BMI, delivery mode, WBC, and PLT. *P* for interaction values are provided for subgroup comparisons.

Multivariable logistic regression analysis demonstrated a significant positive association between HCT and the risk of cranial MRI abnormalities (Table 2). After adjusting for neonatal characteristics, maternal factors, and hematological parameters (Model lll), each 1% increase in HCT was associated with a 6% higher risk of abnormalities (OR = 1.06, 95% CI: 1.01–1.12, *P* = 0.015). When HCT was analyzed by quartiles, neonates in the highest quartile (Q4: 53.8–65.6%) had a 2.4-fold increased risk of abnormalities compared to those in the lowest quartile (Q1: 33.2–45.8%) (OR = 2.39, 95% CI: 1.07–5.35, *P* = 0.034). A significant positive trend was observed across quartiles (*P* for trend = 0.026). The restricted cubic spline analysis confirmed a linear dose-response relationship (*P* for nonlinearity = 0.908), with the risk of abnormalities increasing steadily with higher HCT (Figure 2). Stratified analyses by sex and delivery mode revealed consistent associations, and no significant interactions were detected (*P* for interaction > 0.05). The effect size was slightly more pronounced in male neonates (OR = 1.08, 95% CI: 1.01–1.16) and those delivered by cesarean section (OR = 1.18, 95% CI: 1.00–1.39). Further stratification by maternal GDM did not modify the association (*P* for interaction = 0.436), with consistent effect estimates in non-GDM (OR = 1.08, 95% CI: 1.01–1.14) and GDM subgroups (OR = 1.04, 95% CI: 0.93–1.16) (Table 3, Figure 3).

**Table 2:** Multivariable logistic regression analysis of HCT and the risk of cranial MRI abnormalities in NHB.

Variables	Total N	N (%)	Crude Model *OR* (95% *CI*)	*P*	Model Ⅰ *OR* (95% *CI*)	*P*	Model Ⅱ *OR* (95% *CI*)	*P*	Model Ⅲ *OR* (95% *CI*)	*P*
HCT	410	108 (26.3)	1.05 (1.01∼1.09)	0.020	1.04 (1.00∼1.09)	0.040	1.05 (1.00∼1.10)	0.039	1.06 (1.01∼1.12)	0.015
Q1 33.2%≤HCT < 45.8%	102	19 (18.6)	1 (Ref)		1 (Ref)		1 (Ref)		1 (Ref)	
Q2 45.8%≤HCT < 49.8%	103	26 (25.2)	1.48 (0.76∼2.88)	0.254	1.44 (0.72∼2.91)	0.306	1.61 (0.74∼3.49)	0.230	1.70 (0.78∼3.71)	0.184
Q3 49.8%≤HCT < 53.8%	102	32 (31.4)	2.00 (1.04∼3.83)	0.037	1.93 (0.98∼3.83)	0.059	2.09 (0.97∼4.46)	0.058	2.37 (1.08∼5.20)	0.032
Q4 53.8%≤HCT < 65.6%	103	31 (30.1)	1.88 (0.98∼3.61)	0.058	1.86 (0.92∼3.74)	0.082	2.07 (0.96∼4.49)	0.064	2.39 (1.07∼5.35)	0.034
*P* for trend				0.038		0.057		0.053		0.026

Data are presented as OR and 95% CI.

Crude model, No covariates were adjusted.

Model I, Adjusted for age, sex, birth weight, admission weight, and week of gestation.

Model II, Adjusted for model I and maternal age, pre-pregnancy BMI, and delivery mode.

Model III, Adjusted for model II and for WBC, PLT.

**Table 3:** Stratified analyses of HCT and the risk of cranial MRI abnormalities in NHB.

Subgroup	Total N	N (%)	Crude Model *OR* (95% *CI*)	*P*	Adjusted model *OR* (95% *CI*)	*P*	*P* for interaction
Sex							0.419
Male	227	62 (27.3)	1.07 (1.01∼1.13)	0.015	1.08 (1.01∼1.16)	0.025	
Female	183	46 (25.1)	1.03 (0.97∼1.09)	0.315	1.03 (0.96∼1.11)	0.400	
Delivery mode							0.678
Vaginal delivery	279	96 (34.4)	1.03 (0.99∼1.08)	0.145	1.06 (1.00∼1.11)	0.050	
Cesarean section	125	12 (9.6)	1.08 (0.99∼1.19)	0.095	1.18 (1.00∼1.39)	0.051	
Vaginal delivery to cesarean section	6	0 (0)	1 (0∼Inf)	1.000	1 (0∼Inf)	1.000	
GDM							
No	297	89 (30.0)	1.06 (1.01∼1.10)	0.014	1.08 (1.01∼1.14)	0.015	0.436
Yes	113	19 (16.8)	1.03 (0.94∼1.12)	0.520	1.04 (0.93∼1.16)	0.460	

The adjusted model is Model III: Adjusted for age, sex, birth weight, admission weight, week of gestation, maternal age, pre-pregnancy BMI, delivery mode, WBC, and PLT.

## Discussion

### Summary of key findings

Our study demonstrates a significant linear association between HCT and cranial MRI abnormalities in NHB. The analysis of 410 cases revealed that each 1% increase in HCT was associated with a 6% higher risk of abnormalities (adjusted OR = 1.06, 95% CI: 1.01–1.12), with neonates in the highest HCT quartile showing a 2.4-fold increased risk compared to those in the lowest quartile. These findings remained consistent across multiple adjusted models and were confirmed by restricted cubic spline analysis (*P* for nonlinearity = 0.908). Notably, the robustness of this association was further underscored by stratified analyses, which revealed consistent effects across sex, delivery mode, and GDM, with no significant interactions (*P* > 0.05).

### Comparison with existing literature

Our findings concur with recent studies that underscore the role of hematological factors in neonatal neurological injury while offering significant new insights. A 2022 study by Rosen et al. (12) demonstrated that twin anemia polycythemia sequence (TAPS) was associated with fetal brain lesions, particularly in polycythemic twins, suggesting that higher HCT may contribute to neurological injury. Additionally, Spruijt et al. (13) reported cerebellar hemorrhage in both fetal and neonatal cases of twin-twin transfusion syndrome, further supporting the association between hematological disturbances and brain injury. Consistent with these findings, a study by Rachel Vassar et al. (14) investigated the clinical and imaging characteristics of neonatal polycythemia and brain damage. They found that 45.7% of neonates with polycythemia had brain injuries, with severity correlating with the duration of polycythemia, cerebral oxygen saturation, and abnormal cerebral hemodynamics. This highlights the significant impact of HCT on neonatal brain health and supports our focus on hematological factors in neonatal neurological outcomes. However, our study extends these findings in several key aspects: First, by demonstrating a linear relationship across the entire HCT spectrum rather than just at extreme values; second, through the use of more sensitive and objective MRI-based outcomes; and third, by focusing specifically on the hyperbilirubinemia population, where the interaction between HCT and bilirubin may be particularly relevant.

In contrast to our findings, a 2023 study by Gire et al. (15) reported no significant association between early Hb levels and neurodevelopmental outcomes at two years of age in very preterm infants. This discrepancy may arise from differences in study design and population. Gire et al. specifically examined very preterm infants with a mean gestational age of 28.7 weeks, using Hb levels as the primary hematological parameter, whereas our study evaluated HCT in NHB. Moreover, their study assessed neurodevelopmental outcomes at two years of age using the Ages and Stages Questionnaire (ASQ), while we utilized cranial MRI as a more immediate and objective measure of brain injury. Furthermore, Gire et al.’s study did not specifically focus on the hyperbilirubinemia infants, which may have influenced the observed associations. Similarly, the systematic review by Kalteren et al. (16) highlighted inconsistent correlations between HCT and cerebral oxygenation in preterm infants, with some studies reporting no association. This difference may reflect the complex interplay among anemia, transfusion practices, and gestational age-specific cerebrovascular responses. Importantly, neither of these studies specifically examined the hyperbilirubinemia population, where the combined effects of HCT and bilirubin toxicity might exacerbate neurological risks. Notably, our subgroup analysis of all admitted neonates (Supplementary Table S1) demonstrated that those undergoing MRI exhibited significantly higher bilirubin levels (328.3 vs. 283.9 μmol/L, *P* < 0.001) and longer hospital stays (102.9 vs. 83.5 h, *P* < 0.001), suggesting that these factors may interact with HCT to influence neurological outcomes.

### Dose-response relationship between HCT and cranial abnormalities

In our study, cranial MRI abnormalities were predominantly hemorrhagic lesions (70.4%), followed by ischemic lesions (13.9%), other abnormalities (12.0%), and combined hemorrhagic and ischemic lesions (3.7%) (Supplementary Figure S2). The dose-response relationship between HCT and cranial MRI abnormalities was rigorously evaluated through both continuous and quartile-based analyses (Tables 2, 3). When analyzed as a continuous variable, each 1% increase in HCT was associated with a 6% higher risk of MRI abnormalities in the fully adjusted model (OR: 1.06, 95% CI: 1.01–1.12, *P* = 0.015). More importantly, the quartile analysis revealed a clear biological gradient, with neonates in the higher HCT quartiles (Q3 and Q4) demonstrating significantly increased risks compared to the reference group (Q1), with adjusted ORs of 2.37 (95% CI: 1.08–5.20) and 2.39 (95% CI: 1.07–5.35) respectively. The statistically significant trend test (*P* for trend = 0.026) across fully adjusted models further supports this dose-dependent pattern. Notably, this relationship persisted across progressively adjusted models, with the effect size increasing from Model I to Model III, suggesting that the observed association is independent of potential confounding factors including demographic characteristics, maternal factors, and hematologic parameters. The consistency of this trend across different analytical approaches (continuous vs. categorical) and increasing strength of association with more comprehensive adjustment strengthen the biological plausibility of our findings. Subgroup analyses (Table 3) revealed that this dose-response relationship was particularly evident in male neonates (adjusted OR: 1.08, *P* = 0.025), but was not statistically significant in females. The underlying mechanisms for this differential susceptibility remain unclear. Future studies with larger sample sizes should specifically examine these biological pathways to elucidate the basis for this sex-dependent response. The absence of significant interaction by delivery mode (*P* = 0.678) indicates that the observed association between HCT and cranial MRI abnormalities is robust across different delivery modes. Similarly, stratification by maternal GDM did not modify the association (*P* for interaction = 0.436), further supporting the robustness of the HCT effect. Stratified analyses by abnormality subtype revealed consistent positive trends between HCT and both hemorrhagic (adjusted OR = 1.05, 95% CI: 0.99–1.11) and ischemic lesions (adjusted OR = 1.07, 95% CI: 0.96–1.19), although statistical power was limited by small subgroup sizes (Supplementary Table S3). Future studies with larger sample sizes are warranted to perform subtype-specific analyses.

### Biological mechanisms

The association between elevated HCT and cranial MRI abnormalities in NHB likely involves multiple pathways. Elevated HCT increases blood viscosity, impairing cerebral microcirculation and reducing oxygen delivery (17), as demonstrated by pseudo-continuous arterial spin labeling (pCASL) MRI studies showing an inverse correlation between HCT and cerebral blood flow (18). This hemodynamic compromise may exacerbate bilirubin-induced neurotoxicity, particularly in neonates with immature cerebral autoregulation (19). Additionally, high HCT promotes a pro-inflammatory state characterized by elevated cytokines (e.g., IL-6, IL-8) (20), which may further impair blood-brain barrier function and increase oxidative stress. Bilirubin, in high concentrations, generates reactive oxygen species, further damaging vulnerable neonatal neurons (21, 22). Together, these effects (reduced perfusion, inflammation, and oxidative injury) likely contribute to the observed MRI abnormalities in NHB with elevated HCT.

### Study strengths and limitations

This study has several notable strengths, including a well characterized sample of NHB with standardized cranial MRI assessments, comprehensive adjustment for potential confounders (including maternal, neonatal, and hematological factors), and demonstration of a clear dose-response relationship between HCT and cranial MRI abnormalities. However, the study has several limitations. First, due to its cross-sectional design, we cannot establish temporal relationships or definitively prove causality between HCT and neurological injury. Second, the single center design may limit generalizability to other populations. Third, while we adjusted for multiple potential confounders, residual confounding from unmeasured factors cannot be excluded. Fourth, a significant limitation of this study is the selective performance of cranial MRI based on clinical indications, which may introduce indication bias. Future prospective studies are needed to validate our findings. These limitations warrant careful consideration when interpreting the findings.

## Conclusions

This study found that high levels of HCT were significantly associated with a higher risk of incident cranial MRI abnormalities among NHB. These findings suggest that HCT measurement could direct current risk stratification protocols for neurological complications in this vulnerable population. The results highlight the potential clinical utility of incorporating hematologic parameters into neuroimaging decision making algorithms for NHB. Further multicenter studies are warranted to validate these findings and investigate their implications for clinical management strategies.

## Data Availability

The raw data supporting the conclusions of this article will be made available by the authors, without undue reservation.

## References

[1] Cai Y Li X Wang P Song Y. Predictive factors for readmission due to neonatal hyperbilirubinemia: a retrospective case-control study. PLoS One. (2025) 20:e0320767. 10.1371/journal.pone.0320767 40168352 PMC11960944

[2] van der Meulen NM Meijers KL Dudink J van de Pol LA. Predictive value of brain MRI for neurodevelopmental outcome in infants with severe unconjugated hyperbilirubinemia: a systematic review. Eur J Paediatr Neurol. (2024) 53:49–60. 10.1016/j.ejpn.2024.09.010 39366171

[3] Kim SY Kang HM Im SA Youn YA. The impact of clinical seizures and adverse brain MRI patterns in neonates with hypoxic-ischemic encephalopathy and abnormal neurodevelopment. Clinics (Sao Paulo). (2025) 80:100533. 10.1016/j.clinsp.2024.100533 39752997 PMC11754658

[4] Scholkmann F Ostojic D Isler H Bassler D Wolf M Karen T. Reference ranges for hemoglobin and hematocrit levels in neonates as a function of gestational age (22–42 weeks) and postnatal age (0–29 days): mathematical modeling. Children (Basel). (2019) 6:38. 10.3390/children6030038 30832270 PMC6463180

[5] Sitina M Stark H Schuster S. Optimal hematocrit theory: a review. J Appl Physiol (1985). (2024) 137:494–509. 10.1152/japplphysiol.00034.2024 38813609

[6] Farag MM Thabet M El Beheiry A Hammad B Khalifa MA Elsebaee A. Hemodynamically significant anemia as an indication of transfusion in preterm infants. Ital J Pediatr. (2025) 51:140. 10.1186/s13052-025-01978-w 40380201 PMC12084977

[7] Kuruvilla DJ Widness JA Nalbant D Schmidt RL Mock DM An G Estimation of adult and neonatal RBC lifespans in anemic neonates using RBCs labeled at several discrete biotin densities. Pediatr Res. (2017) 81:905–10. 10.1038/pr.2017.14 28099421 PMC5470643

[8] Pilania RK Saini SS Dutta S Das R Marwaha N Kumar P. Factors affecting efficacy of packed red blood cell transfusion in neonates. Eur J Pediatr. (2017) 176:67–74. 10.1007/s00431-016-2806-7 27864631

[9] Martinez-Biarge M Arnaez J Arca G Valverde E Llorens-Salvador R García-Alix A Recommendations for the use of brain MRI in the neonatal period. An Pediatr (Engl Ed). (2025) 103:503935. 10.1016/j.anpede.2025.503935 41022518

[10] Subspecialty Group of Neonatology tSoP, Chinese Medical A, Editorial Board CJoP. Guidelines on the clinical management of neonatal hyperbilirubinemia (2025). Zhonghua Er Ke Za Zhi. (2025) 63(4):338–50. 10.3760/cma.j.cn112140-20241231-00944 40090910

[11] Jr Harrell FE. Regression Modeling Strategies: With Applications to Linear Models, Logistic Regression, and Survival Analysis. 2nd ed New York: Springer (2015). p. 20–4.

[12] Rosen H Silber R Schwartz A Avnet H Lipitz S Shrot S Fetal and neonatal brain injury in twins complicated by twin anemia polycythemia sequence. Prenat Diagn. (2022) 42(8):978–84. 10.1002/pd.6194 35726441

[13] Spruijt MS van Klink J de Vries LS Slaghekke F Middeldorp JM Lopriore E Fetal and neonatal neuroimaging in twin-twin transfusion syndrome. Ultrasound Obstet Gynecol. (2024) 63(6):746–57. 10.1002/uog.27583 38214436

[14] Vassar R George E Mogga A Li Y Norton ME Glenn O Fetal intraparenchymal hemorrhage imaging patterns, etiology, and outcomes: a single center cohort study. Ann Neurol. (2024) 96:1137–47. 10.1002/ana.27072 39215698 PMC13464407

[15] Gire C Fournier N Pirrello J Marret S Patural H Flamant C Impact of early hemoglobin levels on neurodevelopment outcomes of two-year-olds in very preterm children. Children (Basel). (2023) 10(2):209. 10.3390/children10020209 36832338 PMC9955539

[16] Kalteren WS Verhagen EA Mintzer JP Bos AF Kooi E. Anemia and red blood cell transfusions, cerebral oxygenation, brain injury and development, and neurodevelopmental outcome in preterm infants: a systematic review. Front Pediatr. (2021) 9:644462. 10.3389/fped.2021.644462 33718309 PMC7952449

[17] Roh DJ Murguia-Fuentes R Gurel K Khasiyev F Rahman S Bueno PP Relationships of hematocrit with chronic covert and acute symptomatic lacunar ischemic lesions. Neurology. (2024) 102:e207961. 10.1212/WNL.0000000000207961 38165319 PMC10870744

[18] Ibaraki M Nakamura K Matsubara K Shinohara Y Kinoshita T. Effect of hematocrit on cerebral blood flow measured by pseudo-continuous arterial spin labeling MRI: a comparative study with (15)O-water positron emission tomography. Magn Reson Imaging. (2021) 84:58–68. 10.1016/j.mri.2021.09.012 34562565

[19] Watchko JF Tiribelli C. Bilirubin-induced neurologic damage–mechanisms and management approaches. N Engl J Med. (2013) 369(21):2021–30. 10.1056/NEJMra1308124 24256380

[20] Jain A Deo P Sachdeva MUS Bose P Lad D Prakash G Aberrant expression of cytokines in polycythemia vera correlate with the risk of thrombosis. Blood Cells Mol Dis. (2021) 89:102565. 10.1016/j.bcmd.2021.102565 33831662

[21] Liu C Zhao D Yu G Du HW Xu L Cao Y Alleviation of microglia mediating hippocampal neuron impairments and depression-related behaviors by urolithin B via the SIRT1-FOXO1 pathway. CNS Neurosci Ther. (2025) 31(4):e70379. 10.1111/cns.70379 40237232 PMC12000931

[22] Lee ZM Chang LS Kuo KC Lin MC HR Y. Impact of protein binding capacity and daily dosage of a drug on total Serum bilirubin levels in susceptible infants. Children (Basel). (2023) 10(6):926. 10.3390/children10060926 37371159 PMC10296853

