# Supplementary material for: Association between hematocrit and cranial MRI abnormalities in neonatal hyperbilirubinemia
Source: Front Pediatr. 2026 May 28;14:1785949. doi: 10.3389/fped.2026.1785949 (PMC13253743; doi:10.3389/fped.2026.1785949)
Supplement: Supplementary file 1 [file Datasheet1.pdf]

Title: Association between Hematocrit and Cranial MRI Abnormalities in Neonatal Hyperbilirubinemia

Supplementary Materials

Supplementary figure 1. Distribution of Cranial MRI Results in Neonates with Hyperbilirubinemia.

Supplementary figure 2. Distribution of Cranial MRI Abnormalities in Neonates with Hyperbilirubinemia.

Supplementary table 1. Baseline characteristics of all neonates admitted with hyperbilirubinemia.

Supplementary table 2. Univariate logistic regression analysis of variables and the risk of cranial MRI abnormalities in NHB.

Supplementary figure 1. Distribution of Cranial MRI Results in Neonates with Hyperbilirubinemia.

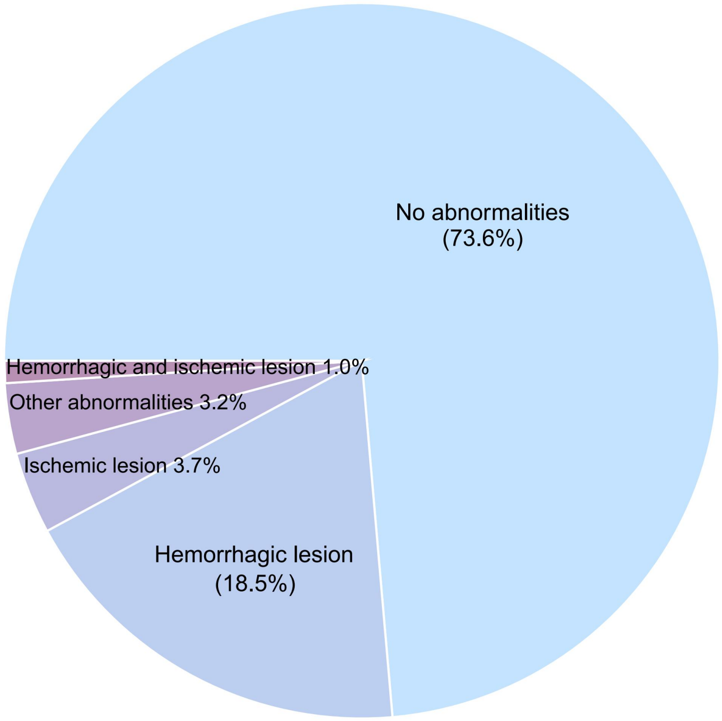

Supplementary figure 2. Distribution of Cranial MRI Abnormalities in Neonates with Hyperbilirubinemia.

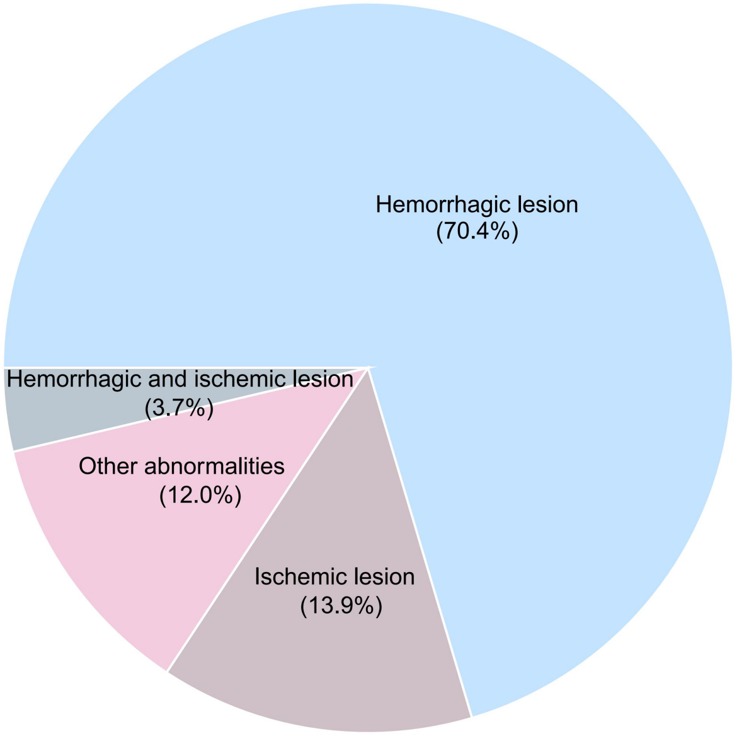

Supplementary table 1. Baseline characteristics of all neonates admitted with hyperbilirubinemia.

Variables	Total (n = 941)	Non-MRI (n = 530)	Undergoing MRI (n = 411)	*P*	Statistic
Diagnosis, n (%)				0.888	0.020
Hyperbilirubinemia	818 (86.9)	460 (86.8)	358 (87.1)		
ABO hemolytic jaundice	123 (13.1)	70 (13.2)	53 (12.9)		
Sex, n (%)				0.464	0.537
Male	507 (53.9)	280 (52.8)	227 (55.2)		
Female	434 (46.1)	250 (47.2)	184 (44.8)		
Birth weight, g	3355.0 ± 422.8	3345.7 ± 404.7	3366.8 ± 445.3	0.448	0.577
Admission weight, g	3203.8 ± 425.4	3216.1 ± 408.1	3187.8 ± 446.9	0.312	1.021
Neonatal blood type, n (%)				0.182	Fisher
O+	268 (28.8)	139 (26.6)	129 (31.5)		
A+	329 (35.3)	192 (36.7)	137 (33.5)		
B+	264 (28.3)	148 (28.3)	116 (28.4)		
AB+	69 (7.4)	44 (8.4)	25 (6.1)		
O-	1 (0.1)	0 (0)	1 (0.2)		
AB-	1 (0.1)	0 (0)	1 (0.2)		
Cranial ultrasonography, n (%)				0.005	Fisher
Subependymal cyst	113 (14.8)	56 (13.3)	57 (16.7)		
Choroidal cyst	32 (4.2)	18 (4.3)	14 (4.1)		
Ventricular enlargement	12 (1.6)	7 (1.7)	5 (1.5)		
Cephalohematoma	13 (1.7)	4 (0.9)	9 (2.6)		
Intracranial hemorrhage	10 (1.3)	0 (0)	10 (2.9)		
Subependymal cyst and cephalohematoma	3 (0.4)	2 (0.5)	1 (0.3)		
Subependymal cyst and choroidal cyst	1 (0.1)	1 (0.2)	0 (0)		
Subependymal cyst and ventricular enlargement	6 (0.8)	3 (0.7)	3 (0.9)		
Subependymal cyst and intracranial hemorrhage	2 (0.3)	1 (0.2)	1 (0.3)		
Choroidal cyst and ventricular enlargement	1 (0.1)	0 (0)	1 (0.3)		
No abnormality	571 (74.7)	330 (78.2)	241 (70.5)		
hsCRP, mg/L	1.1 (0.6, 3.2)	1.0 (0.5, 2.9)	1.2 (0.8, 3.5)	0.004	8.193
RBC, 10^{12}/L	4.9 ± 0.6	4.8 ± 0.6	5.0 ± 0.6	0.002	9.420
HGB, g/L	172.1 ± 20.6	170.5 ± 21.1	174.3 ± 19.9	0.005	7.898
HCT, %	49.3 ± 6.2	49.0 ± 6.4	49.7 ± 6.0	0.078	3.118
MCV, fL	100.7 ± 4.8	101.2 ± 4.7	100.1 ± 4.8	0.001	10.446
MCH, pg	35.2 ± 1.8	35.2 ± 1.8	35.1 ± 1.8	0.388	0.746
MCHC, g/L	349.4 ± 13.6	348.3 ± 13.9	350.9 ± 13.2	0.005	8.052
RDW-CV, %	15.1 ± 1.0	14.9 ± 0.9	15.3 ± 1.1	< 0.001	25.988
RDW-SD, fL	55.3 ± 4.0	55.1 ± 3.9	55.6 ± 4.0	0.061	3.522
WBC, 10^9/L	10.6 ± 3.1	10.5 ± 3.0	10.8 ± 3.3	0.186	1.754
NEUT, 10^9/L	4.8 ± 2.5	4.8 ± 2.4	4.9 ± 2.5	0.271	1.213
LYMPH, 10^9/L	4.2 ± 1.4	4.1 ± 1.4	4.2 ± 1.3	0.452	0.566
MONO, 10^9/L	1.1 ± 0.4	1.1 ± 0.4	1.1 ± 0.4	0.916	0.011
EO, 10^9/L	0.4 (0.3, 0.6)	0.4 (0.3, 0.6)	0.4 (0.3, 0.5)	0.552	0.354
PLT, 10^9/L	287.6 ± 80.4	293.8 ± 79.9	279.7 ± 80.5	0.008	7.125
First TBIL, μmol/L	303.3 ± 51.6	283.9 ± 41.0	328.3 ± 53.2	< 0.001	208.454
First DBIL, μmol/L	5.4 ± 10.3	5.2 ± 10.4	5.8 ± 10.3	0.373	0.796
ALT, U/L	16.4 ± 6.1	16.2 ± 5.9	16.8 ± 6.4	0.172	1.866
AST, U/L	46.0 ± 24.1	44.4 ± 18.5	48.0 ± 29.7	0.023	5.156

TP, g/L	59.5 ± 5.3	59.1 ± 5.1	60.0 ± 5.6	0.009	6.905
ALB, g/L	36.2 ± 3.1	35.7 ± 3.0	36.7 ± 3.2	< 0.001	24.213
GLO, g/L	23.3 ± 3.9	23.4 ± 3.8	23.3 ± 4.0	0.744	0.106
A/G	1.6 ± 0.4	1.6 ± 0.4	1.6 ± 0.4	0.028	4.846
GGT, U/L	152.4 ± 70.9	149.4 ± 73.5	156.1 ± 67.2	0.151	2.065
ALP, U/L	162.1 ± 55.1	161.8 ± 52.7	162.5 ± 58.0	0.841	0.040
GLU, mmol/L	4.2 (3.6, 4.8)	4.3 (3.6, 4.9)	4.1 (3.6, 4.8)	0.103	2.652
Second-TBIL, μmol/L	227.5 ± 46.2	211.9 ± 47.3	237.9 ± 42.5	< 0.001	51.879
Second-DBIL, μmol/L	20.4 ± 7.2	20.7 ± 7.8	20.2 ± 6.7	0.483	0.493
Third-TBIL, μmol/L	140.7 ± 27.8	139.9 ± 26.4	141.5 ± 29.3	0.420	0.650
Third-DBIL, μmol/L	17.1 ± 6.2	16.7 ± 5.2	17.5 ± 7.0	0.085	2.970
Maternal age, year	29.9 ± 4.5	30.1 ± 4.5	29.7 ± 4.4	0.195	1.679
Education, (%)				0.005	18.38
Elementary school	16 (2.0)	11 (2.5)	5 (1.3)		
Junior high school	107 (13.2)	60 (13.8)	47 (12.5)		
High school	58 (7.2)	32 (7.4)	26 (6.9)		
Junior college	82 (10.1)	30 (6.9)	52 (13.9)		
College	251 (31.0)	135 (31.1)	116 (30.9)		
Bachelor	267 (33.0)	144 (33.2)	123 (32.8)		
Master+[a]	28 (3.5)	22 (5.1)	6 (1.6)		
Week of gestation, week	39.0 ± 1.2	39.0 ± 1.2	38.9 ± 1.3	0.111	2.550
Gravidity, n (%)				0.200	7.282
1	438 (46.5)	241 (45.5)	197 (47.9)		
2	222 (23.6)	120 (22.6)	102 (24.8)		
3	143 (15.2)	90 (17.0)	53 (12.9)		
4	72 (7.7)	36 (6.8)	36 (8.8)		
5	42 (4.5)	29 (5.5)	13 (3.2)		
6+[b]	24 (2.6)	14 (2.6)	10 (2.4)		
Parity, n (%)				0.733	Fisher
1	553 (58.8)	311 (58.7)	242 (58.9)		
2	329 (35.0)	181 (34.2)	148 (36.0)		
3	53 (5.6)	34 (6.4)	19 (4.6)		
4	5 (0.5)	3 (0.6)	2 (0.5)		
5	1 (0.1)	1 (0.2)	0 (0)		
Delivery mode, n (%)				0.057	5.716
Vaginal delivery	599 (63.7)	320 (60.4)	279 (67.9)		
Cesarean section	325 (34.5)	199 (37.5)	126 (30.7)		
Vaginal delivery to cesarean section	17 (1.8)	11 (2.1)	6 (1.5)		
Mother blood type, n (%)				0.169	Fisher
O+	356 (40.3)	191 (39.5)	165 (41.1)		
A+	243 (27.5)	147 (30.4)	96 (23.9)		
B+	217 (24.5)	108 (22.4)	109 (27.2)		
AB+	65 (7.4)	36 (7.5)	29 (7.2)		
O-	3 (0.3)	1 (0.2)	2 (0.5)		
Pre-pregnancy BMI, kg/m^2	22.5 ± 3.6	22.4 ± 3.5	22.7 ± 3.7	0.321	0.988
Neonatal age, hour	130.1 ± 81.6	133.4 ± 87.0	125.7 ± 73.9	0.148	2.095
Hospitalization duration, hour	92.0 ± 25.3	83.5 ± 24.4	102.9 ± 22.1	< 0.001	159.360

Notes: Data presented are mean ± standard deviation description, quartile description, or n (%). [a]Master+: Including master's degree and higher. [b]6+: Including pregnancy 6 times and above.

Supplementary table 2. Univariate logistic regression analysis of variables and the risk of cranial MRI abnormalities in NHB.

	OR (95% *CI*)	*P*
Maternal age, years	0.95 (0.90~1.00)	0.043
Delivery mode		
Vaginal delivery	Ref	
Cesarean section	0.20 (0.11~0.39)	<0.001
Vaginal delivery to cesarean section	NA	-
Week of gestation, week	0.90 (0.76~1.06)	0.201
Sex, n (%)		
Male	Ref	
Female	0.89 (0.57~1.39)	0.619
Neonatal age, hour	0.99 (0.99~1.00)	<0.001
Birth weight, g	1.00 (1.00~1.00)	0.829
Admission weight, g	1.00 (1.00~1.00)	0.767
WBC, 10^9/L	0.97 (0.90~1.04)	0.366
PLT, 10^9/L	1.00 (1.00~1.00)	0.158
Pre-pregnancy BMI, kg/m^2	0.95 (0.89~1.02)	0.154
HGB, g/L	1.01 (1.00~1.02)	0.072
RBC, 10^{12}/L	1.42 (0.98~2.04)	0.064
HCT, %	1.05 (1.01~1.09)	0.020
Neonatal blood Type, n (%)		
O+		
A+	0.95 (0.55~1.65)	0.857
B+	1.10 (0.62~1.94)	0.750
AB+	1.35 (0.54~3.43)	0.522
O-	NA	-
AB-	NA	-
Time from birth to first blood collection, hour	0.99 (0.99~1.00)	<0.001
Gravidity, times	0.75 (0.61~0.91)	0.004
Parity, n (%)		
1	Ref	
2	0.53 (0.32~0.86)	0.010
3	0.25 (0.06~1.12)	0.070

Note: "NA" indicates that the data were not available for calculation due to an insufficient number of cases.

Supplementary Table 3 Multivariable logistic regression analysis of HCT and the risk of hemorrhagic lesion in NHB.

	Total N	N (%)	Crude Model *OR* (95% *CI*)	*P*	Adjusted model *OR* (95% *CI*)	*P*
HCT	410	80 (19.5)	1.03 (0.99~1.07)	0.154	1.05 (0.99~1.11)	0.095

Note: The adjusted model is Model III: Adjusted for age, sex, birth weight, admission weight, week of gestation, maternal age, pre-pregnancy BMI, delivery mode, WBC, and PLT.

Supplementary Table 4 Multivariable logistic regression analysis of HCT and the risk of ischemic lesion in NHB.

	Total N	N (%)	Crude Model *OR* (95% *CI*)	*P*	Adjusted model *OR* (95% *CI*)	*P*
HCT	410	19 (4.6)	1.05 (0.97~1.13)	0.27	1.07 (0.96~1.19)	0.197

Note: The adjusted model is Model III: Adjusted for age, sex, birth weight, admission weight, week of gestation, maternal age, pre-pregnancy BMI, delivery mode, WBC, and PLT.
